# Role of Total Body PET/CT in Inflammatory Disorders

**DOI:** 10.1053/j.semnuclmed.2024.11.001

**Published:** 2024-11-21

**Authors:** Shervin Zoghi, Clemens Mingels, Ramsey D. Badawi, Benjamin A. Spencer, Tracy L. Yarbrough, Lorenzo Nardo, Abhijit J. Chaudhari

**Affiliations:** * Department of Radiology, University of California Davis, Sacramento, CA, USA.; † Department of Nuclear Medicine, Inselspital, Bern University Hospital, University of Bern, Bern, Switzerland.

## Abstract

Inflammatory disorders historically have been difficult to monitor with conventional PET imaging due to limitations including radiation exposure, lack of validated imaging biomarkers, low spatial resolution, and long acquisition durations. However, the recent development of long-axial field-of-view (LAFOV) PET/CT scanners may allow utilization of novel noninvasive biomarkers to diagnose, predict outcomes, and monitor therapeutic response of inflammatory conditions. LAFOV PET scanners can image most of the human body (if not the entire body) simultaneously in one bed position, with improved signal collection efficiency compared to conventional PET scanners. This allows for imaging with shorter acquisition durations, decreased injected radiotracer dose, prolonged uptake times, or a combination of any of these. In addition, LAFOV PET scanners enable whole-body dynamic imaging. Altogether, these intrinsically superior capabilities in assessing both local and systemic diseases, have allowed these scanners to make increasingly significant contributions to the assessment of inflammatory conditions. This review aims to further explore the role and benefits of LAFOV scanners for imaging various inflammatory conditions while addressing future developments and challenges faced by this technology.

## Introduction

Standard anatomical imaging modalities such as radiography, CT, and MRI have limitations in assessing pain sources, managing early-stage disease, and monitoring treatment efficacy for inflammatory conditions.^1^ Although tissue contrast plays a role in image utility for such purposes, these techniques have only partially addressed the issues such as early-stage diagnosis and are often unable to adequately assess treatment response.^2^ The mismatch between clinical symptoms and morphological imaging in inflammatory disorders underscores the need for novel biomarkers and imaging, opening the doors to molecular imaging as an emerging modality in this field.

In conjunction with the development of novel biomarkers, the wide-spread implementation of PET scanners has been highly impactful given their unique ability to quantitatively assess disease activity and provide information about the metabolic and molecular activities.^3^ While early PET images were fairly rudimentary compared to those produced today, they were able to address clinical questions which could not be otherwise addressed, such as tissue metabolic changes and glucose consumption in various organs.^4^ Since then, many exceptional improvements have occurred^5–7^; for example, the implementation of iterative reconstruction greatly improved resolution and image quality; the use of CT for attenuation correction simplified the process of obtaining anatomical correlates for metabolic features and reduced costs by shortening scan times; the introduction of time-of-flight acquisition further improved the noise characteristics of the images; finally, in 2019, the clinical implementation of LAFOV scanners played a crucial role in improving the scanner sensitivity leading to improved clinical protocols and vastly expanding the potential clinical and research applications.^8–14^

Unlike short-axial field-of-view (SAFOV) systems, which can only acquire images with a field-of-view of 15–35 cm, LAFOV scanners can capture all the major organs or even the entire patient body in one bed position using abbreviated scan times and lesser doses.^8^ For SAFOV scans to acquire a whole-body PET image (from vertex to toes), continuous bed motion or acquisition of several bed positions with step-and-shoot technique is necessary, leading to an inefficient signal capture of 1–5% because roughly 85–90% of the body is outside the FOV.^15–16^ Furthermore, LAFOV scanners can detect the radiotracer with much greater signal collection efficiency, enabling imaging at later time points to better discriminate between glucose metabolism of malignant cells versus inflammatory processes based on radiotracer kinetics.^17^ Across multiple studies, LAFOV scanners have demonstrated superior image quality with higher spatial resolution, (capable of detecting smaller lesions), more optimal scanning protocols (PET imaging in under 30 seconds or for 5 more half-lives due to the ~40-fold increase in dynamic range), and reduced radiation exposure (dose reduction ratio of ~90%, from 4.5 MBq/kg to 0.57 MBq/kg).^18–24^

The efforts to develop clinical LAFOV scanners have been led by several groups. The first clinically available system was uEXPLORER, a collaboration between University of California, Davis and United Imaging Healthcare on a PET/CT scanner with a 194-cm axial FOV that offers the ability to scan the entire adult body at once.^25–26^ The second clinically available system, Biograph Vision Quadra, was developed by Siemens Healthcare and has a 106 cm axial FOV.^27^ The third is Panorama GS, a scanner developed by United Imaging Healthcare which has become clinically available recently, providing a 148 cm FOV.^28^ Lastly, GE is working on a modular device that will allow scans up to a 128 cm FOV. Several human imaging studies that utilize these systems have been published in inflammatory disorders.^25–27,29–35^

**Figure 1:**
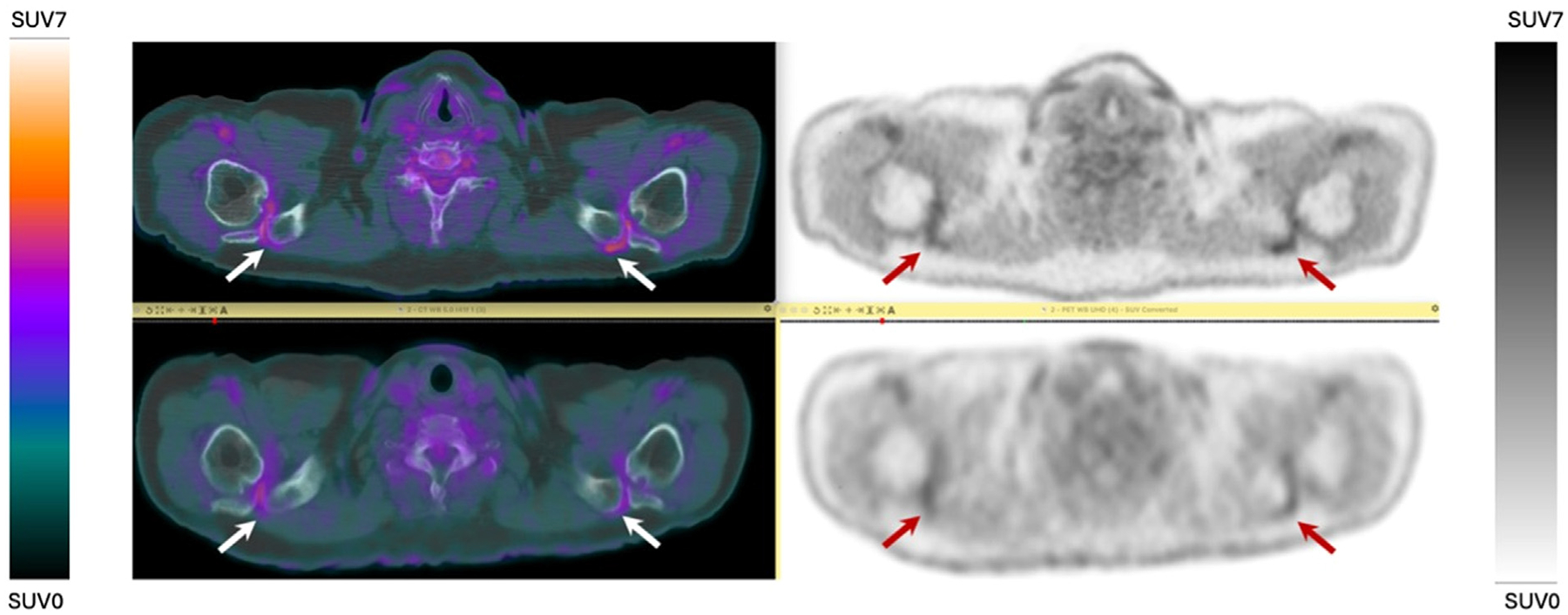
PET/CT of a 72-year-old patient with lung cancer, presented for evaluation of distant metastasis. Axial view images from total-body PET (top) and SAFOV scanner (bottom) were acquired on the same day after an IV injection of 368 MBq [^18^F]FDG. Radiotracer activity is noted at the bilateral acromioclavicular joints. Images obtained on LAFOV scanner demonstrate better spatial resolution and signal-to-noise.

These instrumentation milestones, particularly as it relates to their ability to measure disease metabolism at a molecular level,^36^ have been possible due to the glucose analog radiotracer, 2-deoxy-2-[^18^F]fluoro-D-glucose ([^18^F]FDG). Over the course of the past 40 years, the utilization of [^18^F]FDG-enabled PET imaging^37^ has become the leading tool in assessing the metabolic activity of malignancies, especially in the evaluation of potential recurrence or distant metastasis^38^ (Fig. 1). While it has been used traditionally for oncologic purposes, many studies have also evaluated the utility of [^18^F]FDG PET imaging for the nononcologic musculoskeletal (MSK),^39–41^ pelvic,^42–44^ lymphatic,^45–47^ gastrointestinal,^48–52^ and thoracic^53–55^ systems. Over time, PET has been shown to be a highly effective imaging technique for diagnosing inflammatory conditions, given the active uptake of the [^18^F] FDG in inflammatory cells.^56^ Although the research literature supports the use of [^18^F]FDG PET/CT for inflammatory diseases, several challenges still remain, including the radiation dose, cost, efficacy, and access.^56^ The new generation of LAFOV scanners can address some of these concerns, offering a paradigm shift in imaging towards minimizing dose, optimizing imaging time, and improving the visualization of low-volume disease. The aims of this review article are to explore the role and benefits of LAFOV scanners for imaging various inflammatory conditions and outline future directions and challenges facing this technology.

## Radiotracers

While there are a number of radiotracers available to study inflammation and tumor metabolism with PET, understanding the molecular processes in inflammatory cells can aid our understanding of radiotracer use and breakdown. Glucose transporter proteins, particularly GLUT1 and GLUT3, which are overexpressed in inflammatory and tumor cells, are responsible for the facilitated cellular entry of glucose (or the analogous FDG molecule) and the activation of pro-inflammatory hexokinase and interferon gamma cytokine processes.^57–60^ Secondary effects, such as monocyte activation involving the tyrosine kinase and protein kinase C pathways, along with accumulation of pro-inflammatory M1 macrophages, are further implicated in the increased metabolic activity detected in inflammatory states.^60^ GLUT4, on the other hand, which is primarily expressed in the insulin-sensitive skeletal muscles, can impact global [^18^F]FDG uptake and decrease scan sensitivity by creating additional background noise through elevated signal.^60^ This increase in background noise is seen most frequently among patients who are insulin sensitive or who have recently used their muscles because the increased translocation of GLUT4 to the cell membrane can drastically alter blood glucose distribution.

Given its propensity to accumulate in certain cells because of their high metabolic activity, [^18^F]FDG is FDA approved for a myriad of pathologies including inflammatory disorders, infection of unknown origin, sepsis, vasculitis, and bacteremia,^61^ some of which are explored in the following sections. In 2013, Society of Nuclear Medicine and Molecular Imaging (SNMMI) and the European Association of Nuclear Medicine (EANM) published guidelines supporting [^18^F] FDG’s use in infectious and inflammatory conditions.^62^ Sub-sequently, the Centers for Medicare and Medicaid Services (CMS) retired their noncoverage policy in 2021 and now provide reimbursement for inflammatory and infectious indications.^61^ Due to its established versatility and cost advantage (being more than an order of magnitude less costly than most other radiotracers), [^18^F]FDG continues to be the most commonly utilized radiotracer with both LAFOV or conventional PET/CT scanners.

While [^18^F]FDG is still the most widely used and studied fluorine-based radiotracer, its uptake is not specific to only inflammatory cells, and other fluorinated radiotracers are being explored; these include radiotracers such as [^18^F]florbetapir, [^18^F]florbetaben, and [^18^F]flutemetamol, all of which are being applied to the characterization of the chronic pro-inflammatory neurodegenerative and infiltrative disorders.^63–64^ Many different radiotracers have been developed in the decades since [^18^F]FDG’s debut, albeit with an extremely low number of radiotracers becoming commercially available. For example, radiotracers targeting TSPO, a translocator protein, offer more specificity for imaging of inflammation than [^18^F]FDG, particularly in the brain.^65^ Several compounds based on TSPO are available, demonstrating differences in binding affinity.^66^ For example, Singhal et al demonstrated the utility of using second-generation TSPO ligands, like [^18^F]PBR06, as diagnostic biomarkers for brain inflammation due to their high affinity for innate immune activation in patients with multiple sclerosis.^67^ Despite its utility, however, [^18^F]PBR06 radiotracer is ineffective in approximately 30% of patients due to the rs6971 polymorphism that results in the A147T substitution^68^ which alters the radiotracer binding affinity. Outside the brain, TSPO radiotracers have limitations due to their metabolism and arterial sampling and metabolite correction is essential for quantitative imaging.^69^ UC Davis currently has one IND approved TSPO radiotracer ([^18^F]DPA-714) approved for human use.

Typically used in conjunction with a chelating agent such as desferrioxamine-N-chlorosuccinimide (DBN) or desferrioxamine B (DFO), Zirconium-89 (^89^Zr) is a second radioligand commonly utilized to build new compounds for PET imaging.^70^ Due to its 3.3 day radioactive half-life, ^89^Zr can be used to monitor applications with slow kinetics such as those pertaining to monoclonal antibody-based and anti-inflammatory therapies, and atherosclerotic plaque assessment.^70^ However, because of chelator instability, accumulation in the bone marrow, long half-life, and inefficient signal capture (only 20% of the decay results in positron emission), ^89^Zr compounds possess poor radiochemistry profiles leading to relatively high radiation exposures (~0.54 mSv/MBq, or 20 mSv/mCi).^70–73^ Therefore, the administered activity, and consequently the image quality, are substantially lower than other radioligands such as [^18^F] and thus may necessitate longer acquisition durations and/or improved signal collection efficiency such as that provided by LAFOV. In this setting, the introduction of LAFOV scanners has led to significant improvements in the imaging performance of scanners using these radiotracers.^71^ Before undergoing human clinical studies, Berg et al. conducted a series of experiments using 40 MBq of ^89^Zr labeled radiotracers on nonhuman primates to obtain LAFOV images, with sufficient image quality even up to 30 days (or 9 half-lives) postinjection.^74^ In a study by Mohr et al. comparing LAFOV and SAVOF scans using a ^89^Zr-labelled monoclonal antibody, LAFOV imaging provided significantly lower image noise, ultimately determining that the LAFOV scan duration could be reduced by up to a factor of 8 and still match the image noise of current state-of-the-art SAFOV scanners.^71^ Finally, the reduced dose required when using LAFOV scanners permits protocols which use repeated radiotracer injections, which would be dose-prohibitive with SAFOV scanners.

While the following section on LAFOV imaging for MSK (musculoskeletal) inflammation primarily discusses the commonly utilized [^18^F]FDG radiotracer, existing studies demonstrate the ability of non-FDG PET to image microglia and macrophage activity (DPA-714^75^ and ^18^F-fluoro-PEG-folate^76^), choline metabolism (^11^C-Choline),^77^ cyclooxygenase-2 (COX-2) regulation,^78^ bone turnover (^18^F-NaF),^79–80^ and other aspects of the autoimmune arthritis inflammatory pathway.^81^ Furthermore, the section on LAFOV scanning for vessel inflammation elaborates on the role that [^68^Ga]DOTATATE, an FDA approved radiotracer, plays in monitoring cardiac inflammation.^82^ Despite their promising results, [^18^F]-based radiotracers, such as [^18^F] NaF (microcalcification visualization) and [^18^F]FDG (arterial wall inflammation visualization), provides superior image quality compared to [^68^Ga]-based radiotracers due to their biophysical characteristics including longer half-life (109 vs 68 minutes), shorter positron range (0.27 mm vs 1.05 mm in soft tissue and 0.19 mm vs 0.67 mm in bone tissue), and higher positron yield (96.86% vs 89.14%), respectively.^83–84^

## LAFOV Imaging for MSK Inflammation

Discrepancy between clinical symptoms and findings on conventional radiological imaging findings is common in a myriad of inflammatory conditions; often seen in MSK-related inflammatory conditions, including inflammatory and degenerative arthritis, bursitis, and gout.^85^ These inflammatory conditions are some of the most prevalent MSK conditions and account for the pain, functional limitations, reduced work productivity, and decreased quality of life many patients experience, ultimately resulting in a significant financial burden to society. For example, the cost related to absence from work attributed to chronic pain amounts to approximately $635 billion per year in the United States.^85^ While many patients experience debilitating symptoms, some patients are also asymptomatic and only have evidence of degeneration on MRI only.^86^ The recent introduction of LAFOV scanners offers a unique ability to comprehensively assess disease activity across a host of conditions in symptomatic and, potentially, asymptomatic patients.

Traditionally, assessing the disease burden of arthritis relies heavily on clinical evaluation, laboratory values, and imaging. Given the subjectivity and poor sensitivity and specificity associated with the clinical physical exam,^87–88^ there is a need for standardized markers to enhance early assessment of the disease burden and provide targeted treatment for the specific arthritic subtypes and patient characteristics.^89–91^ One of the most debilitating subtypes of arthritis is Rheumatoid Arthritis (RA). RA is a chronic autoimmune disease characterized by inflammation of the joints and surrounding tissues, as well as variable systemic manifestations involving the lungs, heart, kidneys, and skin.^92^ While conventional radiography and MRI have traditionally been used for detecting the bone, cartilage, and soft tissue inflammatory damage^93^ associated with RA, [^18^F]FDG PET/CT has demonstrated utility in recent years to diagnose, monitor, and treat the systemic inflammation associated with RA, especially in the research setting.^92^

**Figure 2:**
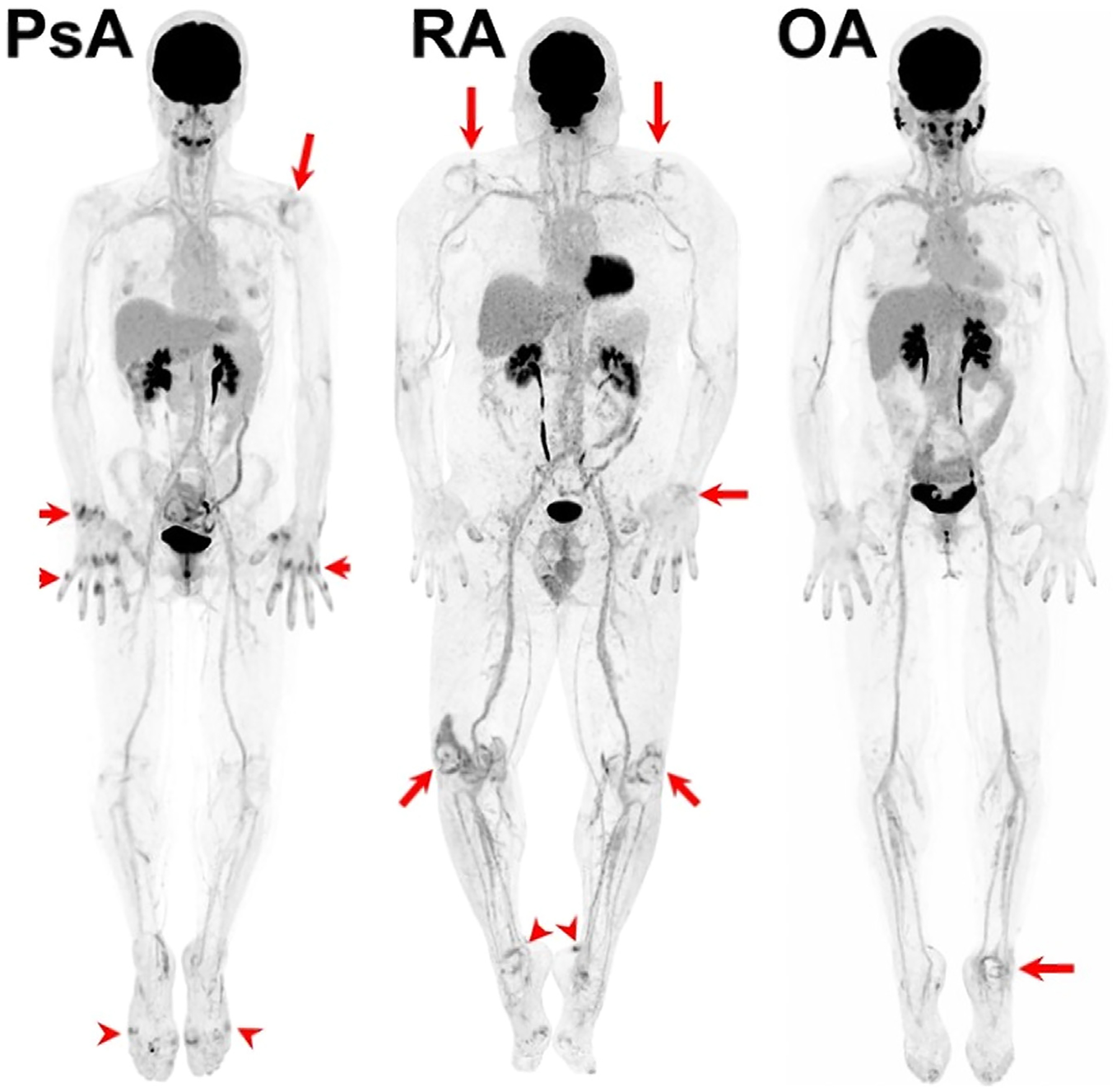
LAFOV imaging of psoriatic arthritis, rheumatoid arthritis, and osteoarthritis.

Clinical trials have demonstrated promising results for monitoring the systemic inflammatory processes associated with RA by detecting metabolic activity, providing a comprehensive whole-body evaluation, and potentially even revealing inflammation prior to observable clinical or structural changes.^94–95^ However, the clinical implementation of PET for arthritic evaluation could not overcome several limitations of the SAFOV scanners including potentially high radiation exposure, limited spatial resolution for small joints, and scan duration.^96^ In this environment, LAFOV scanners have emerged as a powerful tool to overcome these obstacles. A recent study by Abdelhafez et al. demonstrated the capabilities of the LAFOV scanner in visualizing the uptake of [^18^F] FDG at joints across the body in a single scan in patients with autoimmune and nonautoimmune arthritis.^97^ Furthermore, researchers at UC Davis demonstrated that using a LAFOV scanner and an ultra-low dose protocol, it is possible to visualize glucose metabolism in even the smallest joints of the hands and feet.^96^ In the near future, this may open the door to the use of LAFOV scanners in the assessment of subclinical inflammatory joint activity for earlier RA detection while simultaneously monitoring multiple organ systems in order to better understand the systemic changes induced by RA and/or its treatments (Fig. 2).

Psoriatic arthritis (PsA) is another form of autoimmune inflammatory arthritis with potentially devastating systemic complications, including effects on the musculoskeletal, integumentary, vascular, and gastrointestinal systems. Prior studies have established the role of [^18^F]FDG PET/CT for imaging of PsA and components of the downstream inflammatory processes.^96,98^ For instance, a recent study by Kleinrensink et al. highlighted the ability of [^18^F]FDG PET/CT to portray the association between active PsA and aortic vascular inflammation, with a mean aortic target-to-background ratio difference of 1.63±0.17 in subjects with active PsA as opposed to 1.49±0.16 for the control group (*P* ≤ 0.002) when adjusted for gender, age, body mass index, mean arterial pressure and aortic calcification.^98^ However, as is evident in this study where only the aorta was assessed, evaluating a single segment of the body with PET/CT does not yield an adequate picture of the systemic effects of inflammatory conditions and therefore can lead to an incomplete clinical picture. Thus, PsA can commonly be under- or misdiagnosed, leading to ineffective treatments.^99–101^

Many of the limitations of PET/CT imaging in RA are common with PsA and have subsequently served as both model and catalyst for research using LAFOV scanners in PsA. Recent studies demonstrated the characteristic asymmetric joint involvement, uptake, and systemic enthesitis in a single total body scan.^96,102^ In comparison with standardized clinical measures, these LAFOV scans were positive at an additional 16% of joints, 20% of entheses, and 13% of nails that were negative on the standardized assessments, revealing subclinical inflammation that can be useful in the early detection of PsA.^96^ This early identification of PsA, at the point of transition from psoriasis, may allow an earlier commencement of targeted therapeutics, leading to the prevention of painful clinical symptoms and irreversible injury experienced by many patients.

While inflammatory MSK disorders clearly alter glucose uptake and distribution throughout the body, Lu et al. demonstrated that dynamic changes in skeletal glucose metabolism can also be a function of age or metabolic dysregulation.^103^ Additional studies are needed to further assess the potential physiologic mechanisms and implications behind this. However, it will be important for future LAFOV studies to factor in these confounding variables when assessing whole body glucose metabolism.

## LAFOV Imaging for Vessel Inflammation

**Figure 3:**
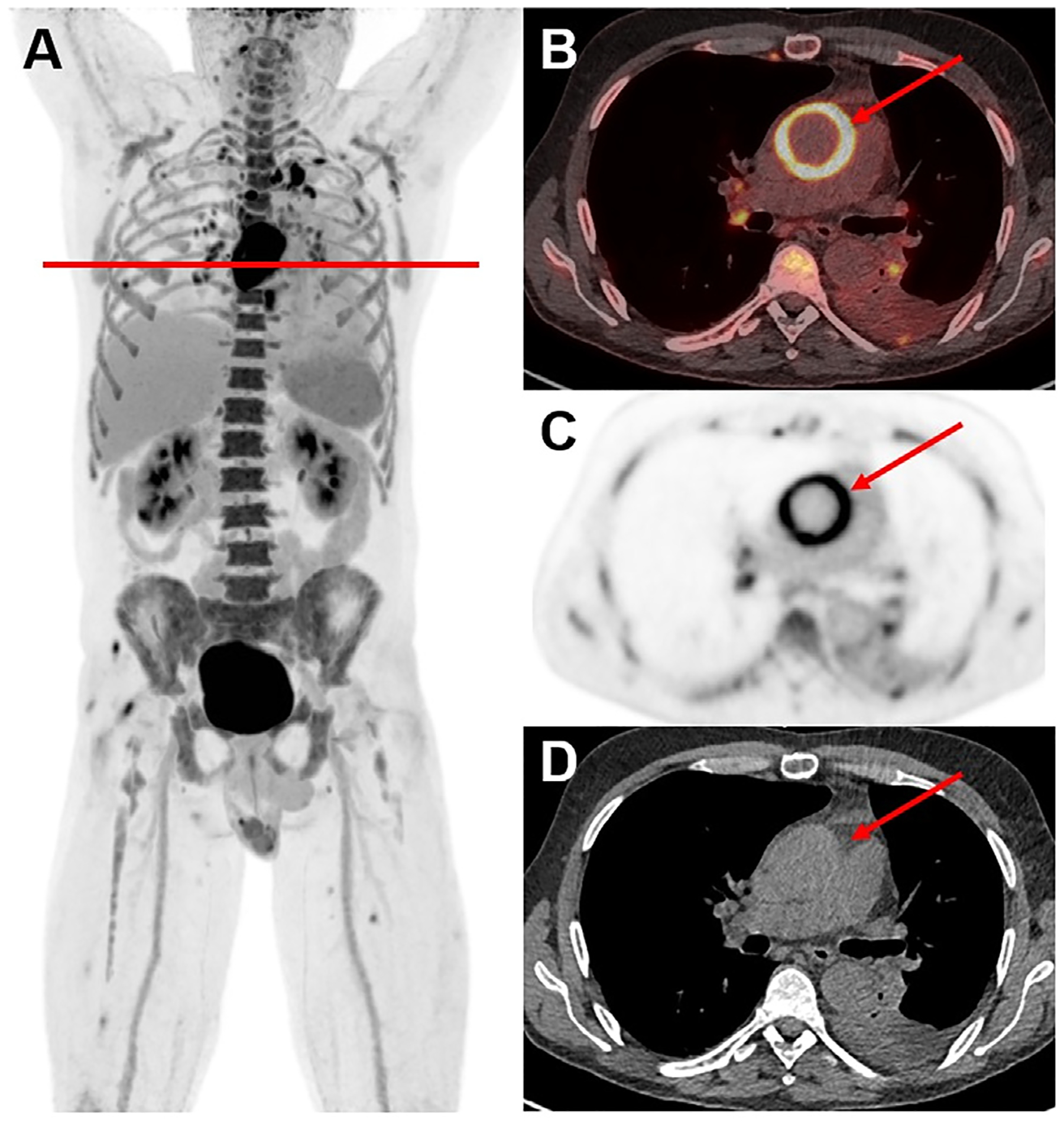
LAFOV PET/CT revealed a graft infection of the ascending aorta in a 59-year-old male patient. Shown are maximum intensity projection (A), LAFOV PET/CT (B), PET-only (C) and CT-only (D) images. The patient suffered from fever of unknown origin with positive blood cultures for streptococcus. LAFOV PET/CT not only revealed the main focus at the ascending aorta (red arrow), but also showed other infectious emboli in the lung and skeletal muscles.

The systemic implications of many inflammatory disorders and diseases such as cancer have a known effect on the vascular system and can lead to inflammation and trans-vascular leakage^104^ Understanding and dynamically modeling these changes has proven challenging, particularly when utilizing SAFOV PET/CT scanners, due to the nature of the arterial wall pathology and lack of large vessels within the FOV (i.e. brain, pelvis, extremities, etc.).^9^ Although PET has been useful in studying arterial wall pathology, the thinness of the wall and its proximity to the blood pool make it challenging to obtain robust signals.^105^ The development of LAFOV scanning has enabled the imaging of large vessel walls with superior quality (Fig. 3), demonstrated in a study by Derlin et al. in which ultra-low dose LAFOV [^18^F]FDG imaging yielded vessel wall signals and target-to-background ratios comparable to those obtained with standard dose SAFOV PET.^105^ The authors concluded that their observations supported the use of LAFOV PET/CT for the detailed assessments of the vessel wall and its interaction with other organs over an extended time after radiotracer use, even at ultra-low doses (~5% of standard dose).^105^

Arteriosclerosis, a condition characterized by the narrowing and hardening of vessel walls, is one of the leading causes of morbidity and mortality worldwide, particularly amongst smokers and older patients.^106^ Commonly recognized for its flow-obstructing implications, which can result in myocardial infarctions and strokes, arteriosclerosis is also known for its fundamental associations with underlying vessel inflammation.^107^ Prior studies have documented the utility of using noninvasive [^18^F]FDG PET for prognosticating coronary events in patients with arteriosclerosis by evaluating levels of active inflammation, plaque vulnerability, and plaque burden.^108^ While this was an important step in the monitoring and treatment of patients with arteriosclerosis, enhancements in PET technology and the development of other radiotracers, such as [^18^F]NaF and [^68^Ga]DOTATATE, has helped enlighten researchers on the mechanistic insights of arteriosclerosis.^109^

LAFOV scanners have also shown significant promise in detecting inflamed coronary plaques in patients with arteriosclerosis and coronary artery disease (CAD). A study by Mingels et al.^83^ demonstrated LAFOV’s superiority over SAFOV in analyzing [^68^Ga]DOTATOC uptake and imaging of calcified plaques. While [^68^Ga]DOTATOC uptake is associated with an increased risk of all-cause mortality and can provide early clues in the detection of inflamed or calcified coronary arteries, it also validates LAFOV PET’s ability to quantify total body calcific activity burden which correlates with systemic arteriosclerosis.^110^ Broader investigations into large vessel vasculitis, cardiac sarcoidosis, and cranial vessel inflammation have also shown significant benefits of LAFOV imaging.^111^

Included in the differential diagnosis of widespread arteriosclerosis is systemic vasculitis, a group of disorders consisting of inflammation in the small, medium, and large vessel walls. Given the recent developments of LAFOV scanning, Lamare et al. were able to differentiate the two disease processes by showing that patients with vasculitis disease have been a greater distribution of inflammatory markers.^112^ Other autoimmune inflammatory vessel wall disorders, such as sarcoidosis and amyloidosis, are further explored in the following section. By providing a more comprehensive and holistic analysis of vascular wall pathology, LAFOV scanners can detect inflammatory disorders earlier and provide more accurate therapeutic options.

## LAFOV Imaging for Systemic Organ System Inflammation

Recent research has revealed that numerous diseases and conditions, previously believed to be isolated to a single organ, involve complex interconnected interactions between multiple organ systems (called connectomes).^113^ For example, one organ system commonly implicated in inflammatory disease processes is the respiratory system. A recent study by Leer et al. looked at COVID-positive acute respiratory distress syndrome (ARDS) patients with persistent lung inflammation.^114^ They determined that LAFOV scanners could achieve superior results with short scanning times (total body scans in 3 minutes) and ultra-low radiotracer doses, making PET/CT imaging more logistically more feasible in critically ill patients.^114^ This is an important finding because ARDS and lung inflammation can originate from systemic processes unrelated to COVID, such as pancreatitis, allergic reactions, autoimmune and inflammatory diseases. Wang *et al*. similarly demonstrated that total-body dynamic [^18^F] FDG PET could detect metabolic changes in multiple organs of the body such as the lungs and spleen in patients recovering COVID-19, signifying a continuation of the inflammatory and immune system responses in the postacute phase.^115^

Assessment of yet another organ potentially involved in systemic inflammation, the liver, can also benefit from the use of LAFOV PET. One of the most common causes of hepatocellular injury – nonalcoholic steatohepatitis – is a chronic pro-inflammatory condition that affects millions of Americans. Despite the ubiquity of this condition, the liver’s dual blood supply makes static hepatic modeling challenging, thus necessitating dynamic noninvasive techniques in order to accurately assess the inflammatory processes in the liver. Wang et al. recently proposed of a dynamic dual-blood input function (DBIF) kinetic model for more accurately quantifying liver inflammation.^116^ Furthermore, a study by Tran et al. found that performing the dynamic imaging and kinetic modeling using LAFOV acquisitions produced excellent assessments of both liver inflammation and steatosis via analysis of the hepatic interstitial space.^117^ These developments have wide-ranging implications for a variety of patient populations given the systemic effects of liver inflammation, including its correlation with renal glucose metabolism and inflammatory biomarkers such as fibroblast growth factor 21 (FGF21).^118^ Although preliminary data in this study focused on the correlation between liver disease severity and kidney glucose metabolism, the implications for altering glucose metabolism in other organs through systemic pro-inflammatory mechanisms and altered hepatokine levels can be further studied using LAFOV scanners in the future.^118^

One such relationship that was further explored by the team at UC Davis was the association between liver inflammation and permeability of the blood brain barrier (BBB) permeability, a process that is implicated in many systemic and neurodegenerative diseases.^119^ This study evaluated the role that high temporal resolution LAFOV scans can play in measuring the molecular-specific permeability of a single radiotracer and depicting the dysregulation in the permeability of the blood brain barrier.^119^ While research is still ongoing for emerging radiotracers and modeling techniques relating to the BBB, PET research on various neuroinflammatory processes has been well-documented. Given the fact that neurodegenerative diseases such as Alzheimer’s disease, multiple sclerosis (MS), and Parkinson’s disease, triggers the neuroinflammatory cascade (inducing oxidative stress and mitochondrial dysfunction), radiotracers like [^18^F]FDG and [^18^F]DPA-714, have emerged as leading diagnostic tools for tracking alterations in the brain physiology.^120^ Furthermore, there are clinical trials currently underway, led by Taswell et al., to assess whether LAFOV scanners can be utilized to improve the evaluation and monitoring of both peripheral and central demyelination in MS.^121^ In this setting, LAFOV scanners particularly beneficial due to their ability to provide a combined assessment of both brain and spinal cord pathologies, revealing the molecular behavior of neurodegenerative diseases like MS that extend beyond the brain into other systems.^122^ This research underscores the unique role that LAFOV PET can play in dynamically obtaining images from interconnected organ systems simultaneously and in further developing noninvasive brain quantification tools for both the research and clinical settings.^123^

Based on these principles, Slart et al. explored the role of LAFOV scanners in studying these multiorgan connections such as the gut-brain or cardio-renal axes.^122^ For instance, utilizing LAFOV scans in heart failure before and during therapy can help researchers and physicians better understanding the interconnected nature of the cardio-renal axis and how different interventions may affect these relationships.^122^ Another example of PET’s ability to assess organ interconnectedness involves the noninvasive quantification of myocardial blood flow and flow reserve, providing unique insights into disease processes, pharmacologic interventions, and more.^124–126^ Moreover, a study by Atsina et al. looked at the activation of extra-cardiac lymphoid organs following cardiac events such as an MI, detecting elevated metabolic activity in the bone marrow and spleen, along with lymph nodes in the axillary, inguinal, and head and neck regions.^137^ In the postacute cardiac event setting, LAFOV imaging has emerged as a powerful tool to elucidate adverse processes early on, paving the way for further studies on multi-organ lymphoid activation.^137^

Cherry et al. assert that examination of these processes which involve the heart primarily and other organs secondarily, as well as systemic disorders that affect the heart and vessels (such as autoimmune diseases like sarcoidosis, amyloidosis, and vasculitis), can benefit from the LAFOV scanner’s ability to capture the cardiovascular system along with the entire body (rather than only the heart as is done with SAFOV scanners).^127^ In patients with sarcoidosis, which is characterized by the development of multi-system inflammation due to the infiltration of noncaseating granulomas, involvement of the heart is associated with significantly elevated morbidity and mortality. LAFOV PET’s ability to demonstrate the characteristic heterogeneous inflammation patterns can aid in the early identification of these pathologies and guide potential biopsy sites for diagnosis.^128–129^ Like sarcoidosis, amyloidosis, which is associated with the deposition of amyloid plaques, can cause an infiltrative cardiomyopathy if left untreated. Several radiotracers, including [^11^C]PiB, [^18^F]florbetapir, and [^18^F]florbetaben, have demonstrated a high affinity for amyloid, including even in cardiac amyloidosis.^129^ In a recent study by Ehman et al., investigators characterized the deposition patterns of light chain amyloidosis using [^18^F]florbetapir across the entire body, and generating SUV distributions for all organs in order to ascertain tissue-specific disease burden.^130^ In that early definitive diagnosis is imperative for superior long-term outcomes in both diseases, these efforts provide support for the potentially high clinical relevance of LAFOV PET-based assessment.

The need to understand the relevance and impact of the gut-heart axis also highlights the opportunities for future avenues of imaging research on lifestyle-associated factors. Currently, the literature on the role of PET/CT in assessing disease activity associated with food-induced inflammation is limited primarily to enteropathy-type T cell lymphoma (a complication of untreated Celiac disease),^131^ studies have been done to establish the feasibility of utilizing [^18^F]FDG to detect sites of active disease for patients with inflammatory bowel disorders (IBD) such as in Crohn’s disease and Ulcerative Colitis.^132–135^ LAFOV PET provides unique opportunities to take this one step further by better monitoring disease activity and flare remissions in patients with IBD in a nonoperative manner.^122^ Furthermore, the prevalence of systemic inflammation associated with certain diets and environments, particularly given their overlap with many of the autoimmune conditions and the gut-body connection discussed earlier in this review, highlights the need for more comprehensive studies on how various diet and environmental factors can be assessed with LAFOV PET/CT. Applying the principles of the connectome model to pathologies involving a number of organ systems can further elucidate novel organ system connections by monitoring the radiotracer dynamics across the entire body at one point in time.^113^ Given the ease and safety of LAFOV scanning, this can lay the foundation for a major paradigm shift in how we pursue disease and treatment monitoring and treatment of other diseases as well.

## Conclusions and Future Directions

While it is clear that the developments in LAFOV scanning are influencing the way we diagnose, monitor, and treat inflammatory conditions, there are still several challenges facing the full clinical adoption of this technology. To unlock its full potential and optimize LAFOV imaging effectively, there is still a need for additional research and confirmatory studies using these scanners on a myriad of inflammatory conditions, dosages, and parameters. Additionally, in conjunction with the increased cost of a LAFOV scanner, robust infra-structure and cutting-edge equipment are required in order to optimize, process, and reconstruct these datasets which contain billions of events and terabytes of data.^10^ Thus, due to the substantial upfront costs currently associated with this technology, it is currently only available to a select few medical centers.

Although promising, decreased costs, broader adoption of the technology, and additional comprehensive studies on other inflammatory disorders and radiotracers are needed before LAFOV PET can become the standard-of-care for imaging and diagnosis of inflammatory disorders.^136^ The development of LAFOV scanners represents a true milestone in the field of medical and molecular imaging, with the potential to change the way we diagnose, monitor, and treat inflammatory conditions, amongst others. While these scanners offer numerous key advantages, including an axial FOV allowing the simultaneous imaging of an entire patient and therefore assess systemic disease activity, and the ability to detect inflammation with higher resolution and lower radiation dose, further studies into the performance of radiotracers and biomarkers are needed to optimally employ these techniques in the diagnosis of a myriad of systemic disease processes, monitor treatment responses, and predict outcomes for various therapies. The existing literature on LAFOV scanners reflects great promise, and has firmly established LAFOV PET’s potential to advance our understanding of systemic inflammatory disease processes.

## Abbreviations:

BBB: Blood brain barrier
CMS: Centers for Medicare and Medicaid Services
CT: Computed Tomography
EANM: European Assoc. of Nuclear Medicine
FDA: Food and Drug Administration
FDG: Fluorodeoxyglucose
Ga: Gallium
GLUT: Glucose transporter
LAFOV: Long axial field of view
MRI: Magnetic Resonance Imaging
MS: Multiple Sclerosis
MSK: Musculoskeletal
PET: Positron Emission Tomography
PsA: Psoriatic Arthritis
RA: Rheumatoid Arthritis
SAFOV: Short axial field of view
SNMMI: Society of Nuclear Medicine and Molecular Imaging
SUV: Standard Uptake Value
TSPO: Translocator protein
Zr: Zirconium
